# Evaluation of serum tRF-23-Q99P9P9NDD as a potential biomarker for the clinical diagnosis of gastric cancer

**DOI:** 10.1186/s10020-022-00491-8

**Published:** 2022-06-11

**Authors:** Yu Zhang, Xinliang Gu, Xinyue Qin, Yuejiao Huang, Shaoqing Ju

**Affiliations:** 1 Medical School of Nantong University, Nantong University, Nantong, China; 2 Department of Laboratory Medicine, Affiliated Hospital of Nantong University, Xisi Road, No. 20, Nantong, China; 3 Research Center of Clinical Medicine, Affiliated Hospital of Nantong University, Nantong, China; 4 Department of Medical Oncology, Affiliated Hospital of Nantong University, Xisi Road, No. 20, Nantong, China

**Keywords:** tRNA-derived small RNAs, tRF-23-Q99P9P9NDD, Gastric cancer, Biomarker, Diagnosis

## Abstract

**Background:**

Gastric cancer (GC) is one of the diseases that endanger human health with high morbidity and mortality. The positive rates of traditional biomarkers in the diagnosis of GC are low, so it is necessary to find biomarkers with high sensitivity to increase the detection rate. tRNA-derived small RNAs (tsRNAs) are novel small non-coding RNAs with specific biological functions and aberrant expression in cancer. In this study, we focused on the potential of tRNA-derived small RNAs as GC biomarkers.

**Methods:**

The differentially expressed tsRNAs in three pairs of GC tissues were screened with high-throughput sequencing and verified using the TCGA database and Quantitative real-time PCR. The methodological evaluation of tRF-23-Q99P9P9NDD was verified by agarose gel electrophoresis, RIN evaluation, and Sanger sequencing. The Chi-square test was used to evaluate the relationship between the tRF-23-Q99P9P9NDD expression and clinicopathological parameters. Kaplan–Meier survival analysis was used to evaluate the effect of the tRF-23-Q99P9P9NDD expression on survival. Additionally, the receiver operating characteristic curve (ROC) was used to evaluate the diagnostic efficacy of tRF-23-Q99P9P9NDD in GC.

**Results:**

Differential expression of serum tRF-23-Q99P9P9NDD could distinguish GC patients from gastritis patients and healthy donors. Chi-square test showed that high expression of tRF-23-Q99P9P9NDD was significantly associated with T stage, lymph node metastasis, TNM stage, and nerve/vascular invasion. Kaplan–Meier curve showed that patients with high expression of tRF-23-Q99P9P9NDD had a lower survival rate than patients with low expression of this biomarker. ROC analysis showed that, compared with conventional biomarkers, the efficacy of tRF-23-Q99P9P9NDD was higher, which was improved by the combination of biomarkers, and even in the early stages. Finally, we preliminarily predicted the downstream of tRF-23-Q99P9P9NDD in GC cells.

**Conclusions:**

The expression of tRF-23-Q99P9P9NDD in GC serum can identify GC patients, and it has higher efficacy than conventional biomarkers even in the early stages. Furthermore, tRF-23-Q99P9P9NDD can monitor the postoperative conditions of GC patients.

**Supplementary Information:**

The online version contains supplementary material available at 10.1186/s10020-022-00491-8.

## Introduction

Gastric cancer (GC) is a common digestive tract malignancy worldwide. The incidence of GC has gradually decreased with increasing research on the eradication of *Helicobacter pylori* infection and changes in dietary structure (Piazuelo and Correa 2013; Wu et al. 2019). Nonetheless, GC remains one of the top three cancers in China (Wang et al. 2019). Most cases of GC are adenocarcinomas, with intestinal adenocarcinomas being more common, and most are located in the gastric antrum and body (den Hoed and Kuipers 2016). The high mortality rate of GC is mainly due to late diagnosis or metastasis of the tumor (Dassen et al. 2010), which results in patients missing the best treatment period and usually having a poor prognosis. The gold standard for GC diagnosis is upper gastrointestinal endoscopy, but this minimally invasive procedure tends to cause patient discomfort. The technique is influenced by the skills of the endoscopist, making diagnostic results relatively variable (Park et al. 2014). Therefore, non-invasive cancer biomarkers released from tumor tissue into the body fluids have attracted attention (Kalniņa et al. 2015; Wang et al. 2020). Current GC biomarkers, including carcinoembryonic antigen (CEA), carbohydrate antigen 199 (CA199), and carbohydrate antigen 724 (CA724), have low positive rates in GC diagnosis (Shimada et al. 2014). Therefore, finding new tumor biomarkers with high sensitivity and specificity is necessary to screen and diagnose GC patients.

A non-coding RNA (ncRNA) is an RNA that does not code for proteins. This class of RNAs has been a hot topic of research and has been shown to play an important role in cancer (Li et al. 2014). It includes long non-coding RNAs (lncRNAs), circular RNAs (circRNAs), pseudogenes, Piwi-interacting RNAs (piRNAs), and microRNAs (miRNAs). Xu et al. identified circRNA_0001178 and circRNA_0000826 as biomarkers of liver metastasis in human colorectal cancer (Xu et al. 2019). According to Chao et al. lncRNA-D16366 could be a promising biomarker for the diagnosis and prognosis of hepatocellular carcinoma (Chao and Zhou 2019). However, these diagnostic biomarkers have certain limitations. Therefore, a search for new biomarkers to assist clinical diagnosis has begun. tRNA-derived small RNAs (tsRNAs) are a new type of non-coding short RNAs that have been gaining popularity. Because tsRNAs have a high abundance and good stability in body fluids, they can become tumor biomarkers (Gu et al. 2021). The biological functions of tsRNAs have been demonstrated to influence tumor cell proliferation, migration, and metastasis (Yu et al. 2020).

tsRNAs, which include tRNA-derived fragments (tRFs) and tRNA halves (tiRNAs), are formed by specialized cleavage of mature tRNAs or pre-tRNAs (Zhu et al. 2019a, b). tRFs, which are 14–30 nucleotides (nt) long and resemble miRNAs with 5′-phosphate and 3′-hydroxyl groups, are attracting increasing interest. tRFs can be divided into five main subclasses, namely, i-tRF, tRF-1, tRF-2, tRF-3, and tRF-5, according to the different digestive sites of Angiogenin (ANG), Dicer, or other RNA enzymes. tiRNAs are usually about 31–40 nt long. Under stressful situations, such as phosphate deprivation, amino acid deficiency, hypoxia, oxidative stress, and viral infection, tiRNAs are generated by the cleavage of anticodon loops of mature tRNAs. 5′tiRNA and 3′tiRNA are the two kinds of tiRNAs (Xie et al. 2020; Zong et al. 2021).

tsRNAs can affect gene expression at both the transcriptional and post-transcriptional stages, with RNA silencing, translation regulation, and epigenetic regulation as the underlying molecular processes (Xie et al. 2020). For example, tRF-3019a forms an RNA-induced silencing complex (RISC) with Ago2 protein, which is directly bound to the 3′UTR of FBXO47 mRNA and degrades mRNA to inhibit translation (Zhang et al. 2020). Under stress, pseudouridylation synthase 7 (PUS7) could bind to tsRNAs and generated mTOG-Ψ8, which is preferentially bound to PABPC1, replacing the translation initiator eIF4G/eIF4E, thus inhibiting the translation (Guzzi et al. 2018). In addition, some tsRNAs could influence the offspring metabolism by regulating epigenetics (Chen et al. 2016). Thus, tsRNAs affect the progress of cancer through these specific molecular mechanisms. For example, Tong et al. demonstrated that tRF-3017A could accelerate GC progression by targeting NELL2 in a manner that resembles miRNA-mediated mRNA silencing (Tong et al. 2020). 5′-tiRNA^Val^, according to Mo et al. targeted FZD3 and thereby suppressed the Wnt/β-Catenin signaling pathway in breast cancer (Mo et al. 2019). In addition, in breast cancer, plasma tRF-Arg-CCT-017, tiRNA-Phe-GAA-003, and tRF-Gly-CCC-001 could be used as novel diagnostic markers and the first two molecules had high prognostic value (Wang et al. 2021). Moreover, tsRNAs may also serve as therapeutic targets for diseases (Sun et al. 2018; Yu et al. 2021), indicating that tsRNAs have increased research value in cancer. Therefore, our team focused on the relationship between the dysregulation of tsRNAs and GC.

High-throughput sequencing technology was used in this study to test for differentially expressed tsRNAs in GC tissues, among which the highly expressed tRF-23-Q99P9P9NDD was selected for further research. We found for the first time that tRF-23-Q99P9P9NDD expression in GC serum was notably higher than normal, and it can be an excellent prognostic biomarker to monitor tumor progression. Furthermore, receiver operating characteristic curve (ROC) analysis revealed that tRF-23-Q99P9P9NDD had higher diagnostic efficacy than CEA, CA199, and CA724, and the diagnostic efficacy was further improved when combined. Therefore, we believe that tRF-23-Q99P9P9NDD has the potential to become a novel biomarker for the diagnosis and prognostic monitoring of GC.

## Materials and methods

### Clinical serum and tissue samples

All of the samples in this study were collected between September 2016 and October 2020, following the ethical guidelines of the World Medical Association. The samples included serum samples from 124 preoperative GC patients, 50 gastritis patients, 119 healthy donors, and 40 postoperative GC patients taken from the Clinical Laboratory of Affiliated Hospital of Nantong University. In addition, 50 pairs of GC tissues and their matched para-cancer tissues collected in the Department of Pathology of our hospital were immediately frozen in liquid nitrogen before being moved to − 80 °C refrigerators for long-term preservation. All of the above patients had their diagnoses confirmed by pathology, did not undergo neoadjuvant radiation, chemotherapy, or targeted therapy, and signed informed consent according to ethical guidelines. The Ethics Committee of Affiliated Hospital of Nantong University has approved this project (ethical review report number: 2018-L055).

### High-throughput sequencing

Trizol was used to isolate total RNA, and RNA quantity and integrity were assessed using Qubit®2.0 and Agilent 2200 TapeStation, respectively. According to the instructions of the manufacturer, 1 μg total RNA of each sample was prepared by NEBNext ® Multiplex Small RNA Library Prep Set for Illumina to prepare a small RNA library. The library was sequenced using a HiSeq 2500 SE50 sequencing system. The clean reads measured were compared with MINTbase using MINTmap software, and new tsRNA prediction was performed for reads not included in MINTbase. EdgeR was used to analyze tsRNAs changes across groups.

### Cell culture

Chinese Academy of Sciences (Shanghai, China) provided human GC cell lines (HGC-27, AGS, MKN-45, SGC-7901, BGC-823) and human gastric mucosal epithelial cell lines (GES-1). All cells were cultured in RPMI 1640 medium (Corning, Manassas, VA, USA) containing 10% fetal bovine serum (Gibco, Waltham, MA, USA), supplemented with 1% penicillin and streptomycin (HyClone, Logan, UT, USA). The culture environment is 37 °C and 5% CO2 humidification incubator.

### Total RNA extraction and cDNA synthesis

A total RNA purification kit and a rotating column separation kit (BioTeke, Wuxi, Jiangsu, China) were used to extract total RNA from the serum of GC patients, and total RNA in tissue and cell samples was extracted using TRIzol Reagent (Invitrogen, Carlsbad, CA, USA). Specific primers (Ribobio, Guangzhou, Guangdong, China) and a Revert Aid RT reverse transcription kit (Thermo Fisher Scientific, Waltham, MA, USA) were used to reverse transcribe total RNA into cDNA in a total of 10 µL incubated for 60 min at 42 °C, which was then inactivated for 5 min at 70 °C. Quantification was required before reaction.

### Quantitative real-time PCR (qRT-PCR)

The qRT-PCR reaction in a total volume of 20 µL was performed on QuantStudio 5 (Thermo Fisher Scientific, Waltham, MA, USA), including 10 µL of ChamQ Universal SYBR qPCR Master Mix (Vazyme Biotech Co., Ltd., Nanjing, Jiangsu, China), 5 µL of cDNA, 1 µL of primers, and 3 µL of enzyme-free water. The primers included forward and reversed primers of tRF-23-Q99P9P9NDD and RNU6B (U6). To standardize the relative expression of tRF-23-Q99P9P9NDD, U6 was used as an internal control. All of the primers were synthesized by RiboBio (Ribobio, Guangzhou, Guangdong, China). Upon the reaction, the data was analyzed using the 2^−ΔΔCt^ method, with the following formula: ΔΔCt = ΔCt_tumor[Ct (target)−Ct (reference)]_ − ΔCt_control[Ct (target)−Ct (reference)]_.

### Nuclear and cytoplasmic RNA isolation assay

Trypsin was used to digest the cultivated MKN-45, HGC-27, and GES-1 cells, and at least 5 × 10^6^ cells were collected into 1.5-mL EP tubes. According to the PARIS™ kit (Thermo Fisher Scientific, Waltham, MA, USA) procedures and instructions of the reagent manufacturer, 60 µL nuclear and cytoplasmic RNA was extracted and then stored in a − 80 °C refrigerator.

### Room temperature and repeated freezing and thawing experiments

Twenty serum samples were randomly mixed and placed at room temperature (25 °C) for 0, 6, 12, 18, and 24 h to extract RNA and detect the expression of tRF-23-Q99P9P9NDD. Subsequently, the mixed serum was freeze-thawed at − 80 °C and room temperature for 0, 1, 3, 5, and 10 times. RNA was extracted and the expression of tRF-23-Q99P9P9NDD was detected.

### Statistical analysis

All of the data in this study were analyzed using SPSS Statistics Version 20.0 (IBM SPSS Statistics, Chicago, IL, USA) and GraphPad Prism 8.0 (GraphPad Software, San Jose, California, USA). The relative expression of tRF-23-Q99P9P9NDD in every group was reported as mean ± Standard Deviation (SD). The corresponding data in this study were first tested for normality using Normality and Lognormality Tests in GraphPad Prism 8.0, and it was found that all of the data did not conform to the normal distribution. Thus, the Mann–Whitney U test was used for comparison of two independent groups, the Wilcoxon matched-pairs signed-rank test was used to analyze the expression levels of tRF-23-Q99P9P9NDD in preoperative and postoperative serum of GC patients, as well as GC tissues and their matched para-cancer tissues, and the Kruskal–Wallis H test was used to compare multiple independent groups. The Chi-square test was used to examine the correlation between tRF-23-Q99P9P9NDD and pathological parameters, and Pearson correlation analysis was used to study other correlations in this work. The Kaplan–Meier curve and the log-rank test were used to assess the survival data. The ROC and Area Under Curve (AUC) were created and computed to assess the diagnostic effect of serum tRF-23-Q99P9P9NDD of GC. The Youden index was used to determine the cut-off value of tRF-23-Q99P9P9NDD, and the cut-off values of CEA, CA199, and CA724 were set according to the reference range of the Affiliated Hospital of Nantong University, which was 5 ng/mL, 37 U/mL, and 10 U/mL, respectively. All of the trials were carried out at least three times independently. When the P-value was < 0.05, the difference was judged to be statistically significant.

## Results

### Expression profiles of tsRNAs in GC and screening of tRF-23-Q99P9P9NDD

**Fig. 1:**
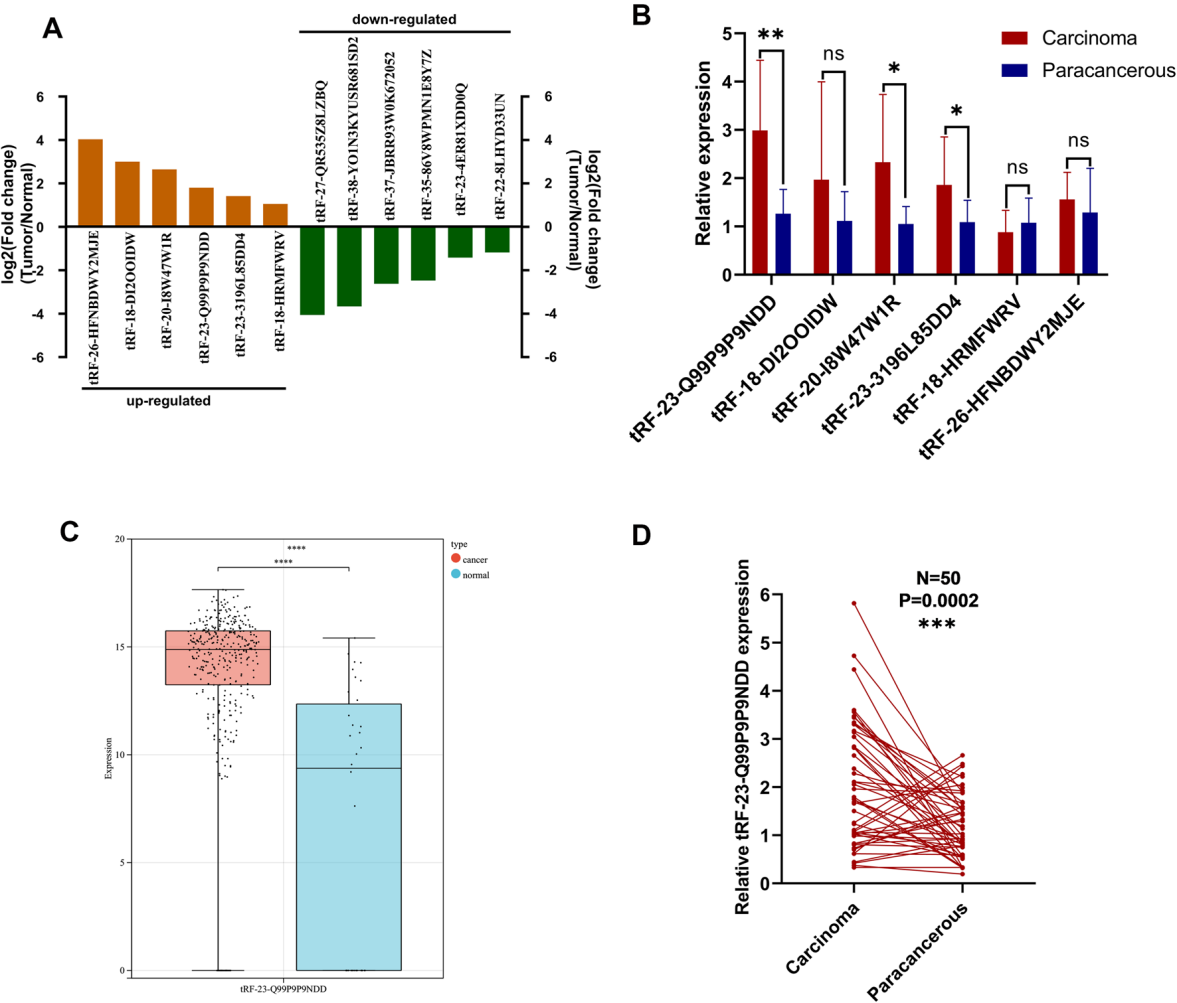
The expression profiles of tsRNAs in GC and the screening of tRF-23-Q99P9P9NDD. **A** Differential expression of tsRNAs-seq in GC tissues and matched para-cancer tissues, with 6 tsRNAs up-regulated (log2 fold change > 1, P < 0.05) and 6 down-regulated (log2 fold change < − 1, P < 0.05). **B** Relative expression of six highly expressed tsRNAs in 10 pairs of GC tissues. **C** Differential expression of tRF-23-Q99P9P9NDD in the TCGA stomach adenocarcinoma database of GC and normal samples. **D** The expression levels of tRF-23-Q99P9P9NDD in 50 pairs of GC tissues and their para-cancer tissues. *P < 0.05; **P < 0.01; ***P < 0.001; ****P < 0.0001

To obtain differentially expressed tsRNAs in GC tissues, we performed high-throughput sequencing on three GC tissues and their matched para-cancer tissues. According to the sequencing results of tsRNAs, we screened six up-regulated (log2 fold change > 1, P < 0.05) and six down-regulated (log2 fold change < − 1, P < 0.05) tsRNAs in tumor tissues between the two groups (Fig. 1A). We performed qRT-PCR on these six highly expressed tsRNAs in 10 pairs of GC tissues, and because the expression level of tRF-23-Q99P9P9NDD was most significantly different (Fig. 1B), it was selected for an in-depth study. We validated tRF-23-Q99P9P9NDD in the TCGA stomach adenocarcinoma database, and we found that the expression of tRF-23-Q99P9P9NDD was significantly higher in GC (Fig. 1C), which is consistent with the sequencing results. In addition, we found that the expression levels of tRF-23-Q99P9P9NDD in GC tissues were significantly higher than in para-cancer tissues through qRT-PCR detection (P = 0.0002) (Fig. 1D).

### tRF-23-Q99P9P9NDD is a sort of tRFs

According to the human genome build (GRCh37/hg19) from the UCSC Genome Browser database (http://genome-asia.ucsc.edu/biomarker.html), tRF-23-Q99P9P9NDD is located on chromosome 6P22.1 with coordinates of 27,248,099–27,248,121 (Additional file 1: Fig. S1A). By checking the basic information of tRF-23-Q99P9P9NDD in MINTbase v2.0 (https://cm.jefferson.edu/MINTbase/) and the OncotRF database (http://bioinformatics.zju.edu.cn/OncotRF/), we identified tRF-23-Q99P9P9NDD as a 5′ tRF (GCTTCTGTAGTGTAGTGGTTATC) of 23 nt in length that was derived from mature tRNA-VAL-CAC, which can be cleaved from tRNA-VAL-CAC-2-1, with the cleavage site located on the D-loop (Additional file 1: Fig. S1B, C). In addition, we found that tRF-23-Q99P9P9NDD had smooth amplification curves and single-peak melting curves (Additional file 1: Fig. S1D, E).

### Methodological evaluation of tRF-23-Q99P9P9NDD

**Fig. 2:**
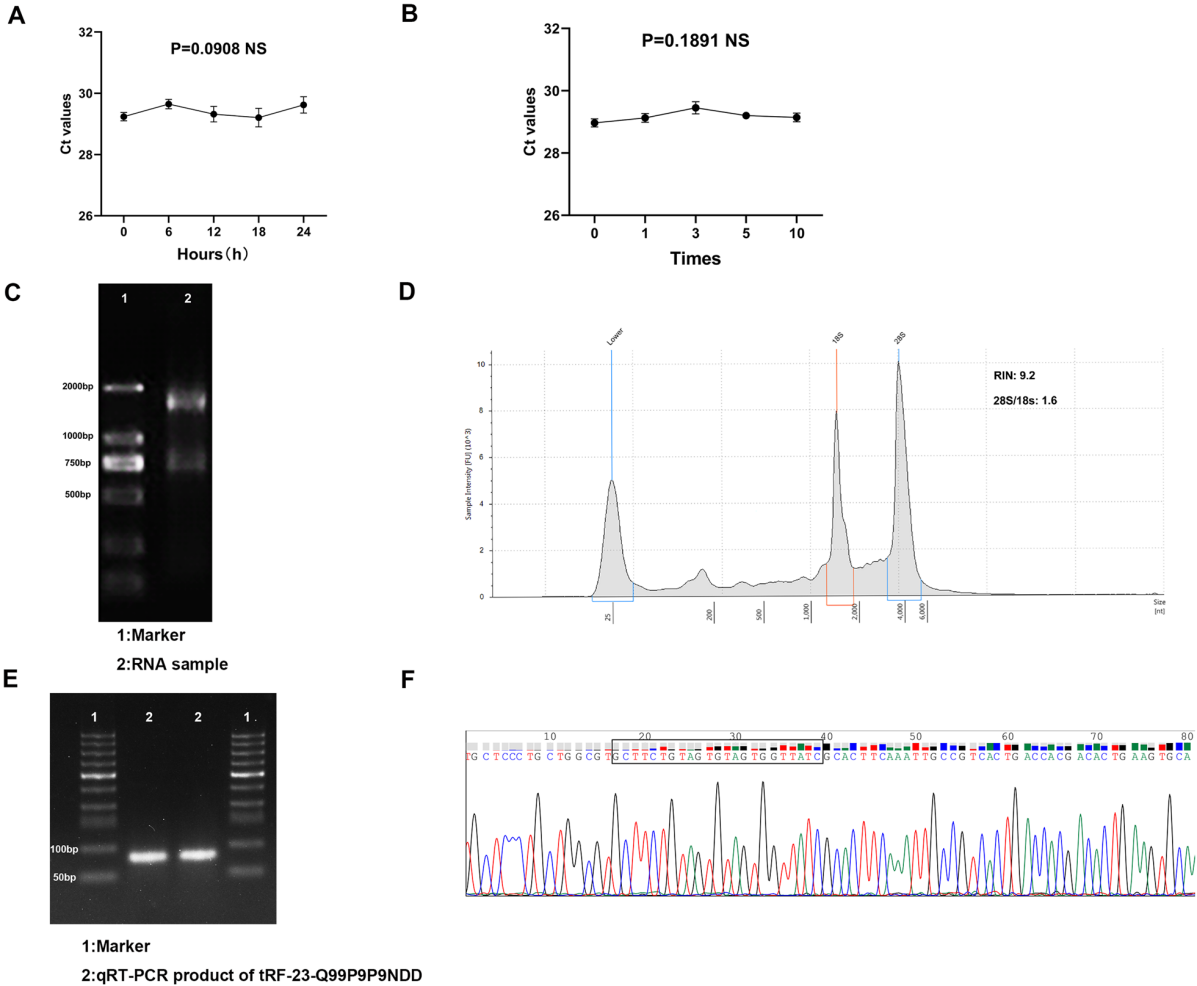
Methodological evaluation of tRF-23-Q99P9P9NDD. **A**, **B** Room temperature placements and repeated freeze–thaw experiments showed no significant changes in the expression level of tRF-23-Q99P9P9NDD. **C** Evaluation of RNA sample integrity by agarose gel electrophoresis. **D** Evaluation of RNA sample integrity by RIN evaluation. **E** The product of qRT-PCR was electrophoresed on 2% agarose gel, and a single band of about 80 bp was obtained. **F** Sanger sequencing of the qRT-PCR product confirmed that the product contained the full-length sequence of tRF-23-Q99P9P9NDD

To investigate whether the method for detecting the expression level of tRF-23-Q99P9P9NDD can be applied in clinical analysis, we performed a comprehensive evaluation. First, we used a mixture of serum to test its accuracy, and we found that tRF-23-Q99P9P9NDD showed a good inter-assay coefficient of variation (CV) and intra-assay CV, which were 2.29% and 1.53%, respectively (Table 1). Then, the mixed serum samples were divided into two groups, which were placed at room temperature for 0, 6, 12, 18, and 24 h, and repeatedly freeze-thawed for 0, 1, 3, 5, and 10 times. The results of the two groups showed that there was no significant difference in tRF-23-Q99P9P9NDD expression with the changes in these conditions (P > 0.05) (Fig. 2A, B), indicating that the detection method of tRF-23-Q99P9P9NDD is not easily affected and has good stability and repeatability. As shown in Fig. 2C, agarose gel electrophoresis (AGE) showed clear bands and RIN evaluation found that the RNA sample had RIN = 9.2, 28S/18S = 1.6, indicating that the RNA sample had good integrity (Fig. 2D). The qRT-PCR product was also detected by AGE, and Fig. 2E shows a single electrophoretic band of about 80 bp and verifies the accuracy of the product. In addition, Sanger sequencing of the product confirmed that it contained the entire tRF-23-Q99P9P9NDD sequence, which was consistent with the sequence of MINTbase v2.0 (Fig. 2F).

**Table 1:** The intra-assay CV and the inter-assay CV of tRF-23-Q99P9P9NDD

	tRF-23-Q99P9P9NDD	U6
Intra assay CV (%)	1.53	2.17
Inter assay CV, (%)	2.29	2.71

*CV* coefficient of variation

### Clinical value and prognostic role of tRF-23-Q99P9P9NDD in GC serum

**Fig. 3:**
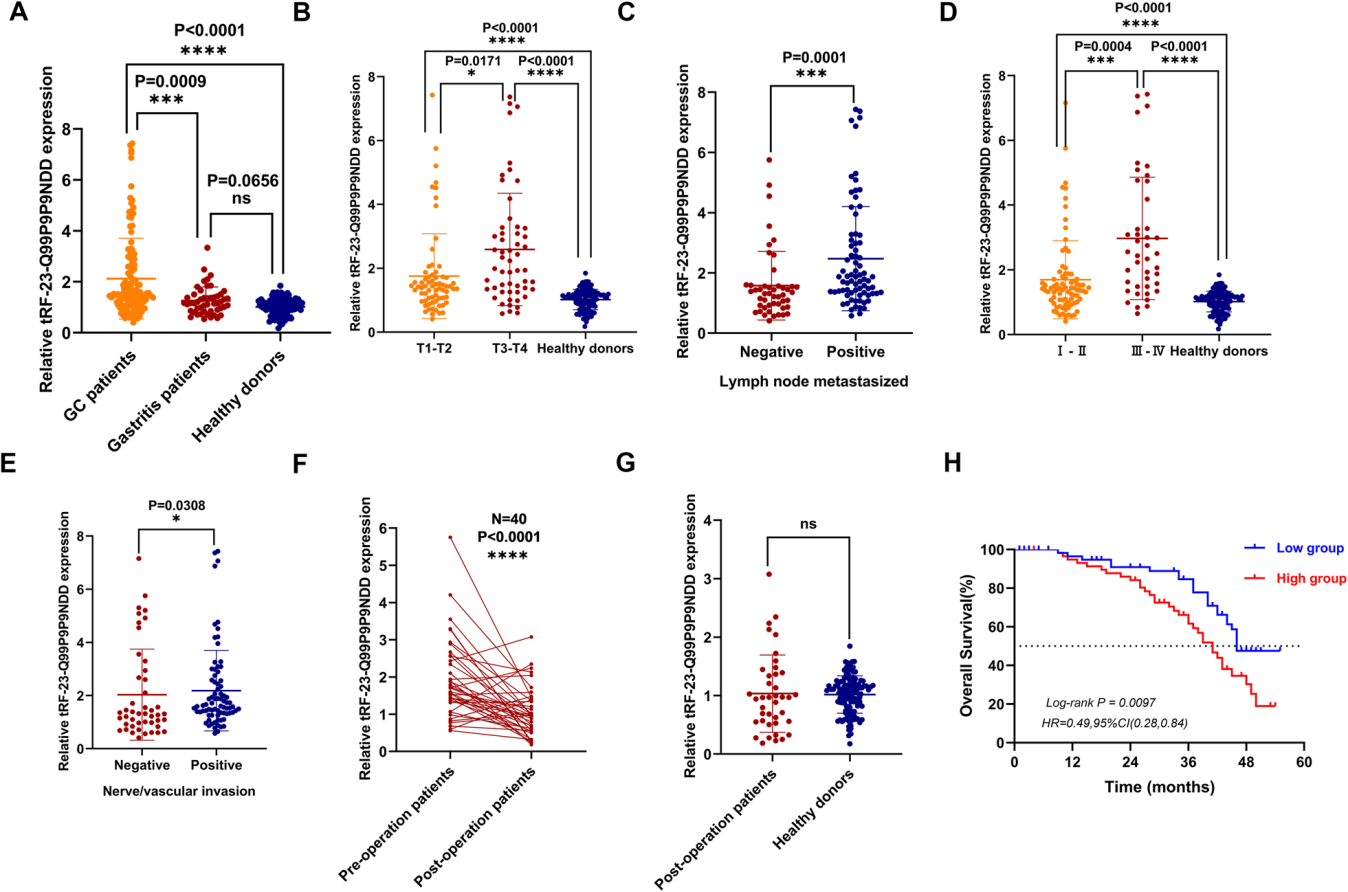
Clinical value and prognostic role of tRF-23-Q99P9P9NDD in GC serum. **A** The expression levels of tRF-23-Q99P9P9NDD in serum samples from GC patients (n = 124), gastritis patients (n = 50), and healthy donors (n = 119). **B** The expression level of tRF-23-Q99P9P9NDD in different stages of the depth of tumor invasion and healthy donors (T1–T2: n = 69, T3–T4: n = 55, healthy donors: n = 119). **C** The expression levels of tRF-23-Q99P9P9NDD in serum of GC patients with (n = 76) or without lymph node metastasis (n = 48). **D** The expression levels of tRF-23-Q99P9P9NDD in serum of stage I–II GC patients (n = 82), stage III–IV patients (n = 42), and healthy donors (n = 119). **E** The expression level of tRF-23-Q99P9P9NDD in serum of GC patients with (n = 78) or without nerve/vascular invasion (n = 46). **F** Changes in the serum expression levels of tRF-23-Q99P9P9NDD in 40 GC patients before and after surgery. **G** Differences in the expression levels of tRF-23-Q99P9P9NDD in the postoperative serum of GC patients and healthy donors. **H** Kaplan–Meier curve analysis of the relationship between the expression levels of tRF-23-Q99P9P9NDD and the survival of GC patients. *P < 0.05; **P < 0.01; ***P < 0.001; ****P < 0.0001

We gathered serum samples from 124 GC patients, 50 gastritis patients, and 119 healthy donors to investigate the diagnostic efficacy of tRF-23-Q99P9P9NDD in the serum of GC patients. The expression level of tRF-23-Q99P9P9NDD in GC patients was substantially higher than that of healthy donors (P < 0.0001) and gastritis patients (P = 0.0009), but the expression level of tRF-23-Q99P9P9NDD in gastritis patients and healthy donors showed no significant difference (P = 0.0656; Fig. 3A). According to the median expression level of tRF-23-Q99P9P9NDD, GC patients were divided into two groups: a relatively high expression group (expression level > 1.519, n = 62) and a relatively low expression group (expression level ≤ 1.519, n = 62), and the Chi-square test was used to evaluate the correlation between tRF-23-Q99P9P9NDD expression and clinicopathological parameters in 124 GC patients. The result showed that the expression level of tRF-23-Q99P9P9NDD was significantly associated with T stage (P = 0.007), lymph node metastasis (P = 0.001), TNM stage (P < 0.0001), and nerve/vascular invasion (P = 0.026), but showed no significant differences with other parameters (sex, age, tumor size, differentiation grade, Lauren classification, C-erbB-2, and MMR) (Table 2), suggesting that the high expression of tRF-23-Q99P9P9NDD may have potential value in predicting the malignancy progression of tumors.

**Table 2:** Clinicopathological analysis of tRF-23-Q99P9P9NDD

Parameter		No. of patients	tRF-23-Q99P9P9NDD (high)	tRF-23-Q99P9P9NDD (low)	P-value
Sex	Male	83	42	41	0.849
	Female	41	20	21	
Age (year)	< 60	39	18	21	0.562
	≥ 60	85	44	41	
Tumor size	< 5	96	50	46	0.39
	≥ 5	28	12	16	
Differentiation grade	Well-maderate	51	27	24	0.584
	Poor-undifferentiation	73	35	38	
T stage	T1-T2	69	27	42	0.007
	T3-T4	55	35	20	
Lymph node metastasis	Positive	76	47	29	0.001
	Negative	48	15	33	
TNM stage	I–II	82	31	51	< 0.0001
	III–IV	42	31	11	
Nerve/vascular invasion	Positive	78	45	33	0.026
	Negative	46	17	29	
Lauren classification	Intestinal type	34	16	18	0.54
	Mixed type	38	17	21	
	Diffuse type	52	29	23	
C-erbB-2	Positive	7	3	4	0.697
	Negative	117	59	58	
MMR	dMMR	3	2	1	0.559
	pMMR	121	60	61	

MLH1, PMS2, MSH2, and MSH6 were all positive for pMMR (normal expression), and 1 or more negative for dMMR (deletion)

Next, the clinicopathological parameters with obvious differences were divided into groups to specifically detect the differences in expression levels of tRF-23-Q99P9P9NDD in each group. The expression level of tRF-23-Q99P9P9NDD increased with the depth of tumor invasion (Fig. 3B). Moreover, lymph node metastasized GC patients had a significantly higher level of tRF-23-Q99P9P9NDD than those of the no-metastasis group (Fig. 3C). Subsequently, we analyzed the expression of tRF-23-Q99P9P9NDD in the serum of healthy donors and stage I–IV GC patients, and we found that the level of tRF-23-Q99P9P9NDD was highest in stage III–IV patients, followed by stage I–II, both of which were significantly higher than in healthy donors (Fig. 3D). Furthermore, the expression level of tRF-23-Q99P9P9NDD in serum of GC patients with nerve/vascular invasion was higher than in GC patients without invasion (Fig. 3E). To verify whether tRF-23-Q99P9P9NDD can be a prognostic biomarker of GC, we tracked the serum tRF-23-Q99P9P9NDD expression in 40 patients with GC after surgery and found that the expression level of tRF-23-Q99P9P9NDD decreased significantly (P < 0.0001) (Fig. 3F) and approached a normal level after surgery (P = 0.2873) (Fig. 3G). Kaplan–Meier analysis showed that the low expression of the tRF-23-Q99P9P9NDD group had a higher survival rate than in the high expression group (Fig. 3H). In conclusion, we reasoned that tRF-23-Q99P9P9NDD has the potential to become a diagnostic biomarker for GC and can dynamically monitor tumor progression.

### Diagnostic value of serum tRF-23-Q99P9P9NDD for GC

**Fig. 4:**
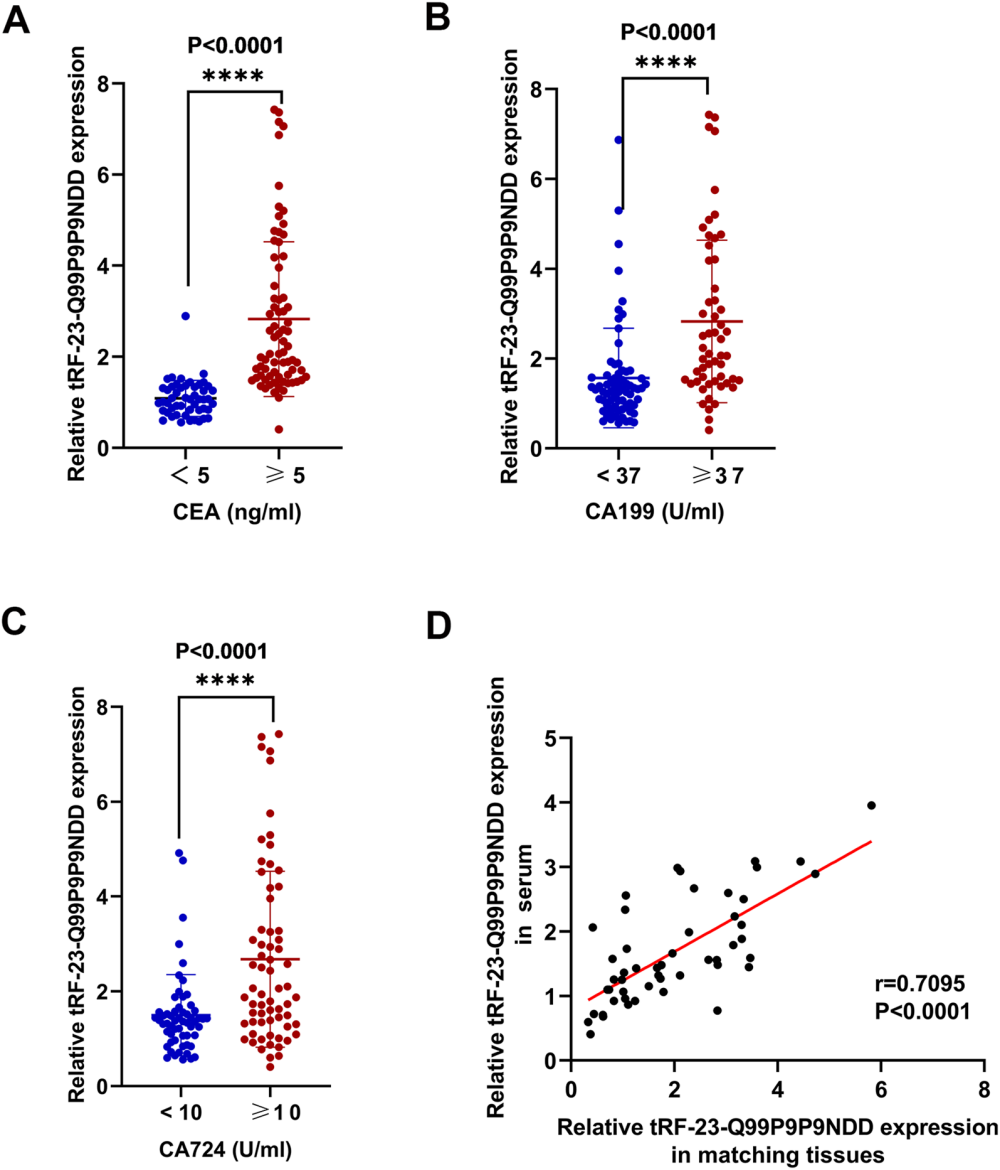
The relationship between the expression levels of serum tRF-23-Q99P9P9NDD and conventional biomarkers of GC. **A–C** GC patients were divided into two groups according to the diagnostic thresholds of CEA, CA199, and CA724, and the expression level of tRF-23-Q99P9P9NDD was analyzed respectively. **D** Pearson correlation analysis was performed on tRF-23-Q99P9P9NDD expression levels in 50 GC tissues and serum samples from corresponding patients. *P < 0.05; **P < 0.01; ***P < 0.001; ****P < 0.0001

CEA, CA199, and CA724 are the most common biomarkers for GC. We discovered that tRF-23-Q99P9P9NDD expression was considerably higher in GC patients with CEA ≥ 5 ng/mL than in patients with CEA < 5 ng/mL (P < 0.0001) (Fig. 4A). Furthermore, GC patients with CA199 ≥ 37 U/mL had a significantly higher level of tRF-23-Q99P9P9NDD than patients with CA199 < 37 U/mL (P < 0.0001) (Fig. 4B). In addition, the tRF-23-Q99P9P9NDD level in GC patients with CA724 ≥ 10 U/mL was significantly higher than in patients with CA724 < 10 U/mL (P < 0.0001) (**Fig. ****4****C)**. Furthermore, the level of tRF-23-Q99P9P9NDD in GC serum was positively correlated with CEA and CA199, but not highly correlated with CA724 (Additional file 2: Fig. S2A-C). We conducted a correlation analysis between 50 GC tissues collected and serum samples of corresponding patients, and the results indicated that patients with higher serum tRF-23-Q99P9P9NDD levels also had higher expression in the paired GC tissues (Fig. 4D).

**Fig. 5:**
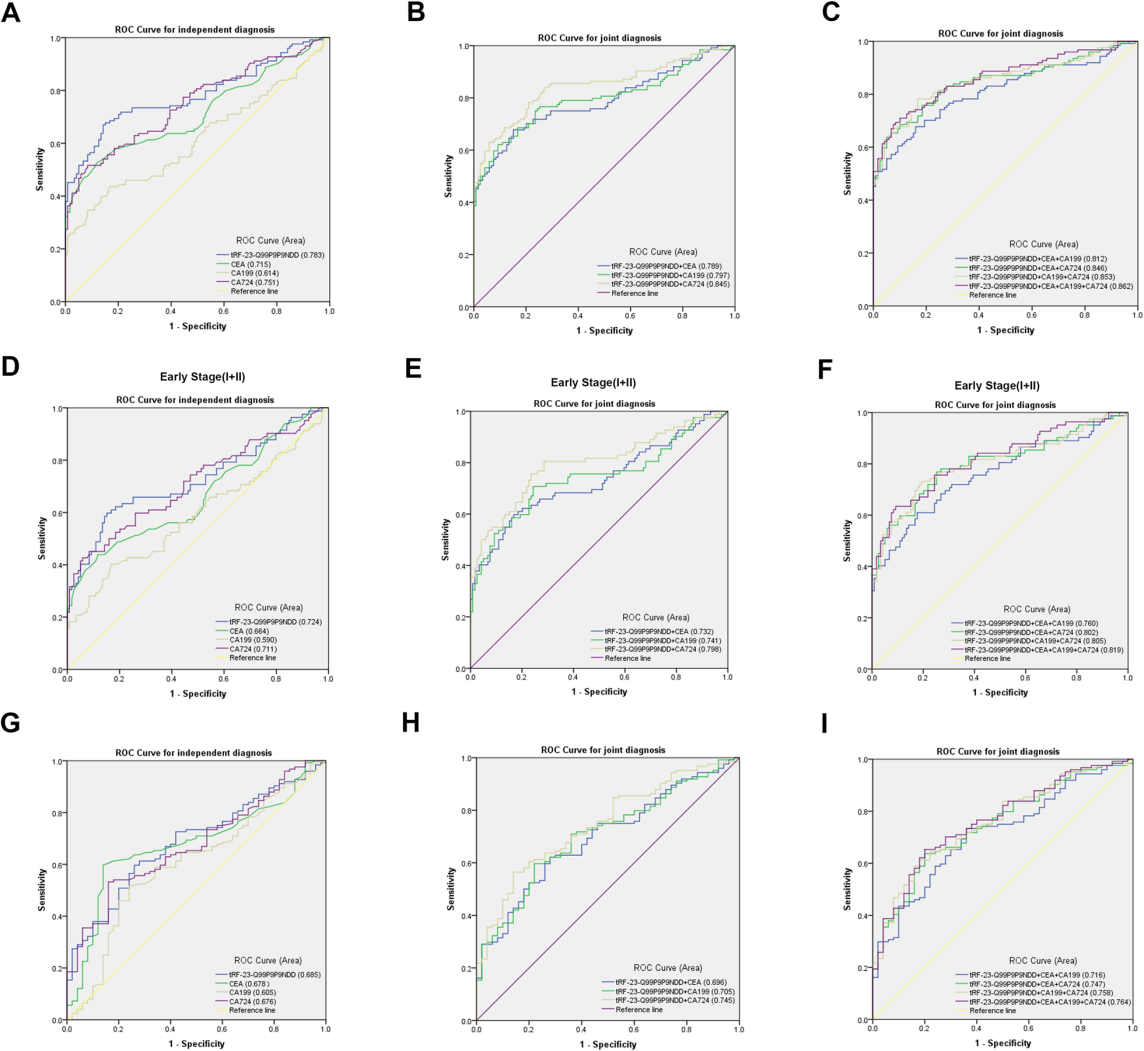
Diagnostic value of serum tRF-23-Q99P9P9NDD for GC. **A**–**C** The diagnostic efficacy of tRF-23-Q99P9P9NDD, CEA, CA199, and CA724 in differentiating GC patients from healthy donors. **D**–**F** The diagnostic efficacy of tRF-23-Q99P9P9NDD, CEA, CA199, and CA724 in differentiating early GC patients from healthy donors was determined by ROC analysis. **G**–**I** The diagnostic efficacy of tRF-23-Q99P9P9NDD, CEA, CA199, and CA724 in differentiating GC patients from gastritis patients was determined by ROC analysis

Next, we comprehensively analyzed the diagnostic efficacy of each biomarker. We performed ROC analyses to determine the diagnostic efficacy of tRF-23-Q99P9P9NDD, CEA, CA199, and CA724 in GC. tRF-23-Q99P9P9NDD had an AUC of 0.783 (95% confidence interval (CI) 0.725–0.842), which was higher than 0.715 (95% CI 0.649–0.780) of CEA, 0.614 (95% CI 0.543–0.686) of CA199, and 0.751 (95% CI 0.690–0.812) of CA724 (Fig. 5A). Meanwhile, tRF-23-Q99P9P9NDD had 67% SEN and 86% SPE in differentiating GC patients from healthy donors when the cut-off point was1.317150645 and the Youden index was 0.526. The SEN of CEA, CA199, and CA724 was 60%, 44%, and 53%, and the SPE was 74%, 77%, and 86%, respectively (Table 3). In addition, the overall accuracy (ACCU), positive predictive value (PPV), and negative predictive value (NPV) of tRF-23-Q99P9P9NDD were as follows: 76%, 83%, and 71%, which were higher than those of CEA, CA199, and CA724. Thus, tRF-23-Q99P9P9NDD demonstrated better diagnostic efficacy than other GC biomarkers when diagnosed alone. Next, tRF-23-Q99P9P9NDD was combined with CEA, CA199, and CA724, as well as the combination of the three and four biomarkers (Fig. 5B, C Additional file 3: Table S1). It was found that the AUC of combined diagnosis was higher than any single biomarker; moreover, the AUC reached the highest value of 0.862, and the SEN also reached the highest value of 83% in the combination of the four biomarkers (Table 3). In conclusion, tRF-23-Q99P9P9NDD may be a potential GC biomarker, and the diagnostic efficacy of a single marker can be improved by the combination of biomarkers.

**Table 3:** The diagnostic performance of tRF-23-Q99P9P9NDD, CEA, CA199 and CA724 in differentiating GC patients from healthy donors

	SEN (%)	SPE (%)	ACCU (%)	PPV (%)	NPV (%)
tRF-23-Q99P9P9NDD	0.67 (83/124)	0.86 (102/119)	0.76 (185/243)	0.83 (83/100)	0.71 (102/143)
CEA	0.60 (74/124)	0.74 (88/119)	0.67 (162/243)	0.70 (74/105)	0.64 (88/138)
CA199	0.44 (55/124)	0.77 (92/119)	0.60 (147/243)	0.67 (55/82)	0.57 (92/161)
CA724	0.53 (66/124)	0.86 (102/119)	0.69 (168/243)	0.80 (66/83)	0.64 (102/160)
tRF-23-Q99P9P9NDD + CEA	0.71 (88/124)	0.65 (77/119)	0.68 (165/243)	0.68 (88/130)	0.68 (77/113)
tRF-23-Q99P9P9NDD + CA199	0.73 (90/124)	0.73 (87/119)	0.73 (177/243)	0.74 (90/122)	0.72 (87/121)
tRF-23-Q99P9P9NDD + CA724	0.80 (99/124)	0.76 (90/119)	0.78 (189/243)	0.77 (99/128)	0.78 (90/115)
tRF-23-Q99P9P9NDD + CEA + CA199	0.75 (93/124)	0.56 (67/119)	0.66 (160/243)	0.64 (93/145)	0.68 (67/98)
tRF-23-Q99P9P9NDD + CEA + CA724	0.81 (101/124)	0.56 (67/119)	0.69 (168/243)	0.66 (101/153)	0.74 (67/90)
tRF-23-Q99P9P9NDD + CEA + CA199 + CA724	0.83 (103/124)	0.49 (58/119)	0.66 (161/243)	0.63 (103/164)	0.73 (58/79)

*SEN* sensitivity; *SPE* specificity; *ACCU* overall accuracy; *PPV* positive predictive value; *NPV* negative predictive value

Due to the low SEN of clinical biomarkers, most GC patients are already at an intermediate to an advanced stage by the time they are diagnosed, missing the best chance for early treatment. We collected the information from 82 early GC patients (stages I and II) and 119 healthy donors for evaluation. The ROC curves showed that the AUC of tRF-23-Q99P9P9NDD was 0.724 (95% CI 0.648–0.800), which was better than 0.664 (95% CI 0.584–0.743) of CEA, 0.590 (95% CI 0.506–0.673) of CA199, and 0.711 (95% CI 0.636–0.787) of CA724 (Fig. 5D). In addition, the SEN of tRF-23-Q99P9P9NDD was 60%, the SPE was 85% when the cut-off point was 1.309670707, and the Youden index was 0.446, and the ACCU, PPV, and NPV were 75%, 73%, and 75%, respectively, which were all higher than those of CEA, CA199, and CA724. It was satisfactory that the AUC of combined diagnosis was higher than that of a single biomarker in identifying early GC patients and healthy donors (Fig. 5E, F, Additional file 4: Table S2). The AUC reached the highest when the four were combined, which was 0.819, and the SEN increased to 78% (Table 4). The above findings indicate the potential value of tRF-23-Q99P9P9NDD for the early diagnosis of GC.

**Table 4:** The diagnostic performance of tRF-23-Q99P9P9NDD, CEA, CA199 and CA724 in differentiating early GC patients from healthy donors

	SEN (%)	SPE (%)	ACCU (%)	PPV (%)	NPV (%)
tRF-23-Q99P9P9NDD	0.60 (49/82)	0.85 (101/119)	0.75 (150/201)	0.73 (49/67)	0.75 (101/134)
CEA	0.51 (42/82)	0.74 (88/119)	0.65 (130/201)	0.58 (42/73)	0.69 (88/128)
CA199	0.41 (34/82)	0.77 (92/119)	0.63 (126/201)	0.56 (34/61)	0.66 (92/140)
CA724	0.46 (38/82)	0.86 (102/119)	0.70 (140/201)	0.69 (38/55)	0.70 (102/146)
tRF-23-Q99P9P9NDD + CEA	0.65 (53/82)	0.64 (76/119)	0.64 (129/201)	0.55 (53/96)	0.72 (76/105)
tRF-23-Q99P9P9NDD + CA199	0.66 (54/82)	0.72 (86/119)	0.70 (140/201)	0.62 (54/87)	0.75 (86/114)
tRF-23-Q99P9P9NDD + CA724	0.74 (61/82)	0.75 (89/119)	0.75 (150/201)	0.67 (61/91)	0.81 (89/110)
tRF-23-Q99P9P9NDD + CEA + CA199	0.68 (56/82)	0.55 (66/119)	0.61 (122/201)	0.51 (56/109)	0.72 (66/92)
tRF-23-Q99P9P9NDD + CEA + CA724	0.77 (63/82)	0.55 (66/119)	0.64 (129/201)	0.54 (63/116)	0.78 (66/85)
tRF-23-Q99P9P9NDD + CEA + CA199 + CA724	0.78 (64/82)	0.48 (57/119)	0.60 (121/201)	0.51 (64/126)	0.76 (57/75)

*SEN* sensitivity; *SPE* specificity; *ACCU* overall accuracy; *PPV* positive predictive value; *NPV* negative predictive value

It is well known that GC may be transformed from chronic gastritis (Sipponen and Maaroos 2015), and in this study, the expression levels of tRF-23-Q99P9P9NDD in the serum of GC and gastritis patients are different. Thus, we performed a ROC analysis on the data of 124 GC patients and 50 gastritis patients, and we discovered that the AUC of tRF-23-Q99P9P9NDD was 0.685 (95% CI 0.605–0.766), which was higher than the 0.678 (95% CI 0.594–0.761) for CEA, 0.605 (95% CI 0.512–0.697) for CA199, and 0.676 (95% CI 0.594–0.757) for CA724 (Fig. 5G). tRF-23-Q99P9P9NDD had 60% SEN and 74% SPE in differentiating GC patients from healthy donors when the cut-off point was 1.391759517 and the Youden index was 0.337. When tRF-23-Q99P9P9NDD was combined with other biomarkers, the AUC increased, reaching the highest AUC of 0.764 (95% CI0.690–0.837) when all four biomarkers were combined (Fig. 5H, I, Additional file 5: Table S3), and the SEN increased to 81% (Table 5).

**Table 5:** The diagnostic performance of tRF-23-Q99P9P9NDD, CEA, CA199 and CA724 in differentiating GC patients from gastritis patients

	SEN (%)	SPE (%)	ACCU (%)	PPV (%)	NPV (%)
tRF-23-Q99P9P9NDD	0.60 (74/124)	0.74 (37/50)	0.64 (111/174)	0.85 (74/87)	0.43 (37/87)
CEA	0.60 (74/124)	0.86 (43/50)	0.67 (117/174)	0.91 (74/81)	0.46 (43/93)
CA199	0.44 (55/124)	0.80 (40/50)	0.55 (95/174)	0.85 (55/65)	0.37 (40/109)
CA724	0.53 (66/124)	0.84 (42/50)	0.62 (108/174)	0.89 (66/74)	0.42 (42/100)
tRF-23-Q99P9P9NDD + CEA	0.66 (82/124)	0.68 (34/50)	0.67 (116/174)	0.84 (82/98)	0.45 (34/76)
tRF-23-Q99P9P9NDD + CA199	0.67 (83/124)	0.60 (30/50)	0.65 (113/174)	0.81 (83/103)	0.42 (30/71)
tRF-23-Q99P9P9NDD + CA724	0.76 (94/124)	0.66 (33/50)	0.73 (127/174)	0.85 (94/111)	0.52 (33/63)
tRF-23-Q99P9P9NDD + CEA + CA199	0.70 (87/124)	0.56 (28/50)	0.66 (115/174)	0.80 (87/109)	0.43 (28/65)
tRF-23-Q99P9P9NDD + CEA + CA724	0.80 (99/124)	0.60 (30/50)	0.74 (129/174)	0.83 (99/119)	0.55 (30/55)
tRF-23-Q99P9P9NDD + CEA + CA199 + CA724	0.81 (101/124)	0.48 (24/50)	0.72 (125/174)	0.80 (101/127)	0.51 (24/47)

*SEN* sensitivity; *SPE* specificity; *ACCU* overall accuracy; *PPV* positive predictive value; *NPV* negative predictive value

### Prediction of tRF-23-Q99P9P9NDD downstream in GC cells

**Fig. 6:**
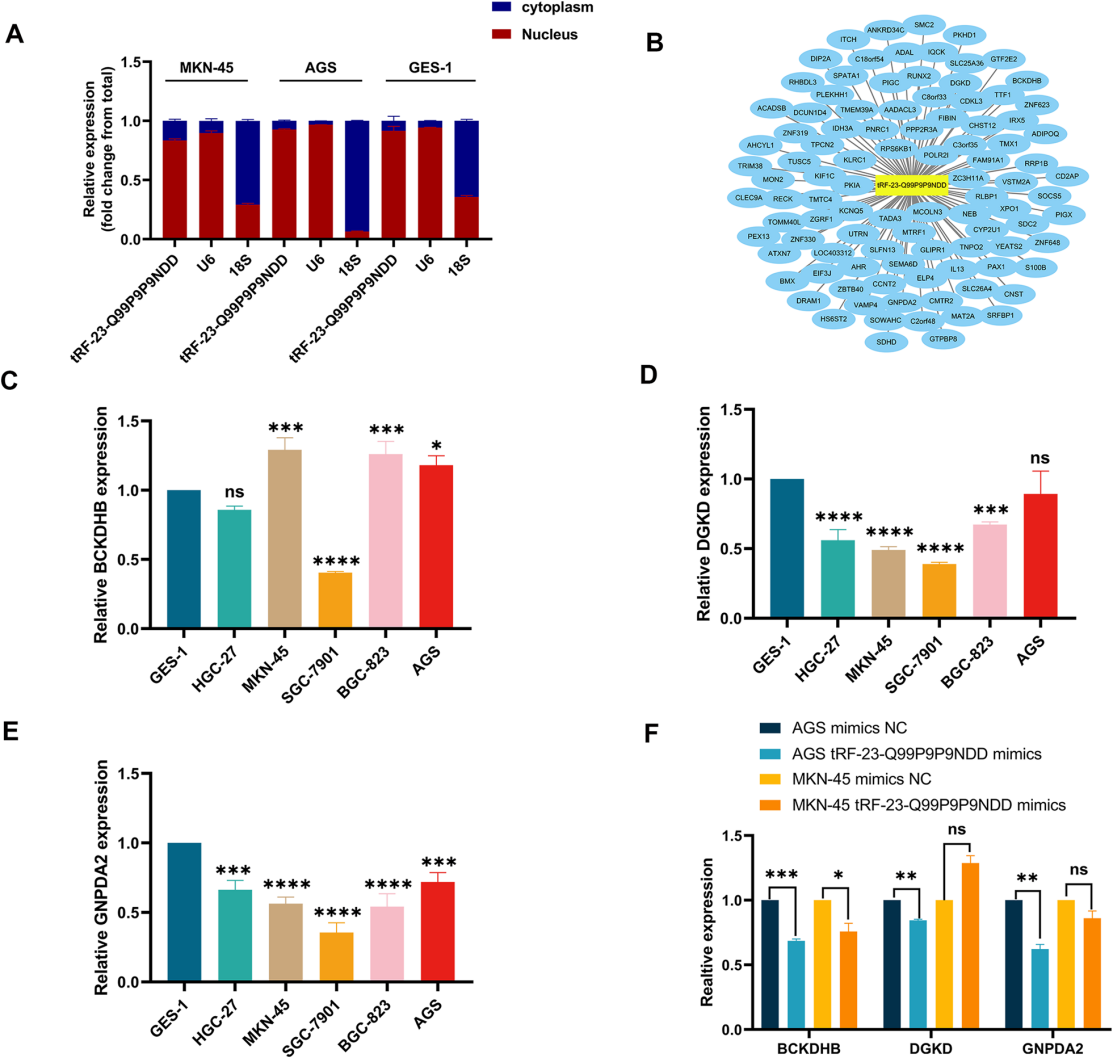
Prediction of tRF-23-Q99P9P9NDD downstream in GC cells.** A** Nuclear and Cytoplasmic RNA Separation Assay was performed on MKN-45, AGS, and GES-1 for the detection of tRF-23-Q99P9P9NDD. **B** Potential target genes of tRF-23-Q99P9P9NDD. **C** The expression levels of BCKDHB in GC cell lines.** D** The expression levels of DGKD in GC cell lines.** E** The expression levels of GNPDA2 in GC cell lines. **F** The expression levels of three target genes in GC cell lines after transfection of tRF-23-Q99P9P9NDD mimics and mimics NC

The cellular localization of tRF-23-Q99P9P9NDD was detected by nuclear and cytoplasmic RNA separation assays, showing that tRF-23-Q99P9P9NDD was mainly located in the nucleus, which provided some ideas for our future experiments (Fig. 6A). Because the function of tRF-23-Q99P9P9NDD may be similar to that of miRNAs, we used bioinformatics database analysis (miRanda, RNAhybrid, TargetScan) to predict the potential mechanism of tsRNAs-mRNA regulatory axis in GC. The connection network showed 100 targeted genes corresponding to tRF-23-Q99P9P9NDD (Fig. 6B). Subsequently, we verified the expression levels of three potential target genes (*BCKDHB*, *DGKD*, and *GNPDA2*) in GC cell lines (Fig. 6C–E). After transfection of tRF-23-Q99P9P9NDD mimics and mimics NC into AGS and MKN-45, the expression trends of these three target genes were verified (Fig. 6F). These results indicated that tRF-23-Q99P9P9NDD may play an important role in the occurrence and development of GC by targeting downstream mRNA, which provides a new direction for further exploration of specific molecular mechanisms of tRF-23-Q99P9P9NDD in GC.

## Discussion

GC is one of the most critical cancers in the world. Although surgical techniques, radiotherapy, and chemotherapy drugs have progressed, the morbidity and mortality of GC still rank fifth and fourth worldwide, respectively (Tan 2019; Sung et al. 2021). Early GC patients usually have no apparent symptoms, and 80% of GC patients are at the late stage when diagnosed, which leads to their missed opportunity for radical resection (Wang et al. 2019). However, because the SEN and SPE of existing clinical tumor biomarkers are not high enough (Wu et al. 2014), we need to find new biomarkers to improve the early diagnosis value of GC. With the development of high-throughput sequencing technology, it has been found that the analysis of differential gene expression can help discover potential cancer biomarkers and advance human research on the mechanism of tumor disease (Govindarajan et al. 2020; Hong et al. 2020).

Through high-throughput sequencing, the gene regulatory role of ncRNA in cancer has been further revealed, and its application as a cancer biomarker has gradually become widespread (Esteller 2011; Sun and Chen 2020). However, the existing tumor biomarkers still have certain limitations. Therefore, researchers still keep finding more efficient tumor biomarkers. When short RNAs less than 40 nt in length for research were selected, it was discovered that many sequencing reads were mapped to RNA fragments produced by the cleavage of tRNA transcripts, known as tsRNAs (Romano et al. 2017). tsRNAs are a new class of small ncRNA produced by enzymatic cleavage of tRNAs and are stably present with specific biological functions in serum, saliva, and extracellular vesicles, among other locations (Dhahbi et al. 2013; Anderson and Ivanov 2014; Chiou et al. 2018; Li et al. 2018; Sun and Chen 2020). More and more cases of abnormal tsRNAs expression in cancer have been discovered. In non-small cell lung cancer, for example, tRF‐Leu‐CAG is highly elevated (Shao et al. 2017). Similarly, patients with liver cancer have much higher levels of tRF-5^GluCTC^ in their plasma exosomes than healthy donors, which can be a new biomarker for diagnosing liver cancer (Zhu et al. 2019a, b). In this study, tRF-23-Q99P9P9NDD with high expression in GC was screened out by high-throughput sequencing. To explore the diagnostic value of tRF-23-Q99P9P9NDD in GC, we expanded the sample size for verification. The results showed that tRF-23-Q99P9P9NDD expression in GC serum was indeed higher than in gastritis patients and healthy donors, which could clearly distinguish GC patients from gastritis patients and healthy donors. Perhaps more promisingly, tsRNAs could also be used to monitor disease progression, not just for the diagnosis. We found that the expression level of serum tRF-23-Q99P9P9NDD was significantly decreased after surgery. In addition, analysis of clinicopathological parameters indicated that the expression level of tRF-23-Q99P9P9NDD was positively associated with T stage, lymph node metastasis, TNM stage, and nerve/vascular invasion, implying that it could be a potential GC therapeutic target. According to the Kaplan–Meier analysis, decreased expression group was associated with a more prolonged overall survival. All of these studies indicated that tRF-23-Q99P9P9NDD has great promise as a clinical biomarker for GC diagnosis and prognosis.

Through different regulatory mechanisms, tsRNAs can influence the process of tumorigenesis and progression (Anderson and Ivanov 2014; Schimmel 2018; Zhu et al. 2019a, b; Yu et al. 2020). For example, Xu et al. established that tRF-Glu-TTC-027 could inhibit the progress of GC by inhibiting the MAPK signaling pathway (Xu et al. 2021). By targeting FBXO47, Zhang et al. found that tRF-3019a promoted GC cell proliferation, migration, and invasion (Zhang et al. 2020). They focused on exploring the regulatory mechanism of tsRNAs in cancer, and only briefly described the expression of molecules and preliminarily evaluated the diagnostic efficacy without specifically analyzing the diagnostic value of tsRNAs as tumor markers. This study explored the potential of tRF-23-Q99P9P9NDD as a GC biomarker for the first time. First, we evaluated the detection method of tRF-23-Q99P9P9NDD and found that its performance was relatively stable, which shows promise for clinical application. Next, we comprehensively analyzed the diagnostic efficacy of tRF-23-Q99P9P9NDD, and we found that the difference in the expression level of serum tRF-23-Q99P9P9NDD could help us identify GC patients and monitor GC progression. Importantly, when we compared tRF-23-Q99P9P9NDD with the clinical GC biomarkers CEA, CA199, and CA724 by ROC, we found that tRF-23-Q99P9P9NDD had higher diagnostic efficiency than other biomarkers, and the combination further improved the diagnostic efficiency. A point of caution is that many current biomarkers are not easily detected in the early stages; they are usually detected in the late stages of GC (Necula et al. 2019). Therefore, we evaluated the diagnostic efficacy of tRF-23-Q99P9P9NDD in the early stages (stages I and II). It is satisfactory that the AUC and sensitivity of tRF-23-Q99P9P9NDD were higher than those of clinical common biomarkers. Thus, tRF-23-Q99P9P9NDD can better distinguish early GC from healthy donors, and a new strategy for early GC diagnosis based on this biomarker should be developed soon. However, our samples did not cover multiple regional specimens due to the limited conditions. The experimental results may not be representative and the sample size still needs to be supplemented. Next, we will continue to collect specimens and data for further analysis to confirm the diagnostic efficiency of tRF-23-Q99P9P9NDD in GC.

Although some progress has been made in our study, the underlying mechanism of differential expression of tRF-23-Q99P9P9NDD remains unclear. With the proliferation of tumor cells, local tumor cells are in a hypoxia state. Tao et al. found that under the regulation of the hypoxia-inducible factor 1 subunit alpha (HIF1α)/ANG axis, 5′tiRNA-His-GTG increased, which promotes the proliferation of colorectal cancer cells by inhibiting the Hippo signaling pathway (Tao et al. 2021). In addition, Cui et al. demonstrated that hypoxia-induced tsRNAs also play an essential role in breast cancer (Goodarzi et al. 2015; Cui et al. 2019). Therefore, it is reasonable to speculate that the generation of tRF-23-Q99P9P9NDD is associated with a hypoxic environment. Furthermore, in this study, we found that tRF-23-Q99P9P9NDD belongs to 5′tRF, which is consistent with the analysis of Kumar et al. thus indicating that 5′tRF mainly exists in the nucleus (Kumar et al. 2016). According to previous studies, tsRNAs may promote or inhibit cancer through different internal mechanisms. Therefore, we speculated that tRF-23-Q99P9P9NDD might be involved in post-transcriptional regulation through a miRNA-like role, promoting GC progression (Sun et al. 2018; Yu et al. 2021). Then, we predicted the potential target genes of tRF-23-Q99P9P9NDD through the database and preliminarily addressed the mechanism.

## Conclusions

We concluded, for the first time, that tRF-23-Q99P9P9NDD is highly expressed in GC cells, tissues, and serum, and it may serve as a potential biomarker for GC. tRF-23-Q99P9P9NDD has higher diagnostic efficacy than conventional biomarkers even in the early stage. Meanwhile, the expression of tRF-23-Q99P9P9NDD was correlated with the progression of GC. We will further study the mechanism of tRF-23-Q99P9P9NDD in GC in the future.

## Supplementary Information

**Additional file 1: Fig. S1. **tRF-23-Q99P9P9NDD is a sortof tRFs.** A **UCSC Genome Browser database showed that tRF-23-Q99P9P9NDDwas located on chromosome 6p22.1 with coordinates 27,248,099–27,248,121.** B **InMINTbase v2.0, tRF-23-Q99P9P9NDD is a 5′tRF (GCTTCTGTAGTGTAGTGGTTATC) with alength of 23nt. **C** OncotRF database showed that the cleavage site waslocated on the D-loop.** D **Amplification curves of tRF-23-Q99P9P9NDD.** E**Melting curves of tRF-23-Q99P9P9NDD.

**Additional file 2: Fig. S2. **The correlation analysis between the expression levels of serumtRF-23-Q99P9P9NDD and conventional biomarkers of GC.** A-C** Pearsoncorrelation analysis was performed on the expression level of tRF-23-Q99P9P9NDDand the levels of CEA, CA199, and CA724 in GC serum.

**Additional file 3: Table S1. **ROC analysis of allbiomarkers in distinguishing GC patients from healthy donors.

**Additional file 4: Table S2.** ROC analysis of allbiomarkers in distinguishing early GC patients from healthy donors.

**Additional file 5: Table S3.** ROC analysis of all biomarkers indistinguishing GC patients from gastritis patients.

## Data Availability

Data are available upon reasonable request. The data used in the current study are available from the corresponding author on reasonable request.

## Abbreviations

GC: Gastric cancer
CEA: Carcinoembryonic antigen
CA199: Carbohydrate antigen199
CA724: Carbohydrate antigen724
lncRNAs: Long non-coding RNAs
circRNAs: Circular RNAs
piRNAs: Piwi-interacting RNAs
miRNAs: MicroRNAs
tsRNAs: TRNA-derived small RNAs
tRFs: TRNA-derived fragments
tiRNAs: TRNA halves
nt: Nucleotides
ANG: Angiogenin
RISC: RNA-induced silencing complex
PUS7: Pseudouridylation synthase 7
qRT-PCR: Quantitative real-time PCR
ROC: Receiver operating characteristic curve
SD: Standard deviation
AUC: Area under curve
SEN: Sensitivity
SPE: Specificity
AGE: Agarose gel electrophoresis
CV: Coefficient of variation
CI: Confidence interval
ACCU: The overall accuracy
PPV: Positive predictive value
NPV: Negative predictive value
