# Supplementary figures and images for: Evaluation of serum tRF-23-Q99P9P9NDD as a potential biomarker for the clinical diagnosis of gastric cancer
Source: Mol Med. 2022 Jun 11;28:63. doi: 10.1186/s10020-022-00491-8 (PMC9188071; doi:10.1186/s10020-022-00491-8)

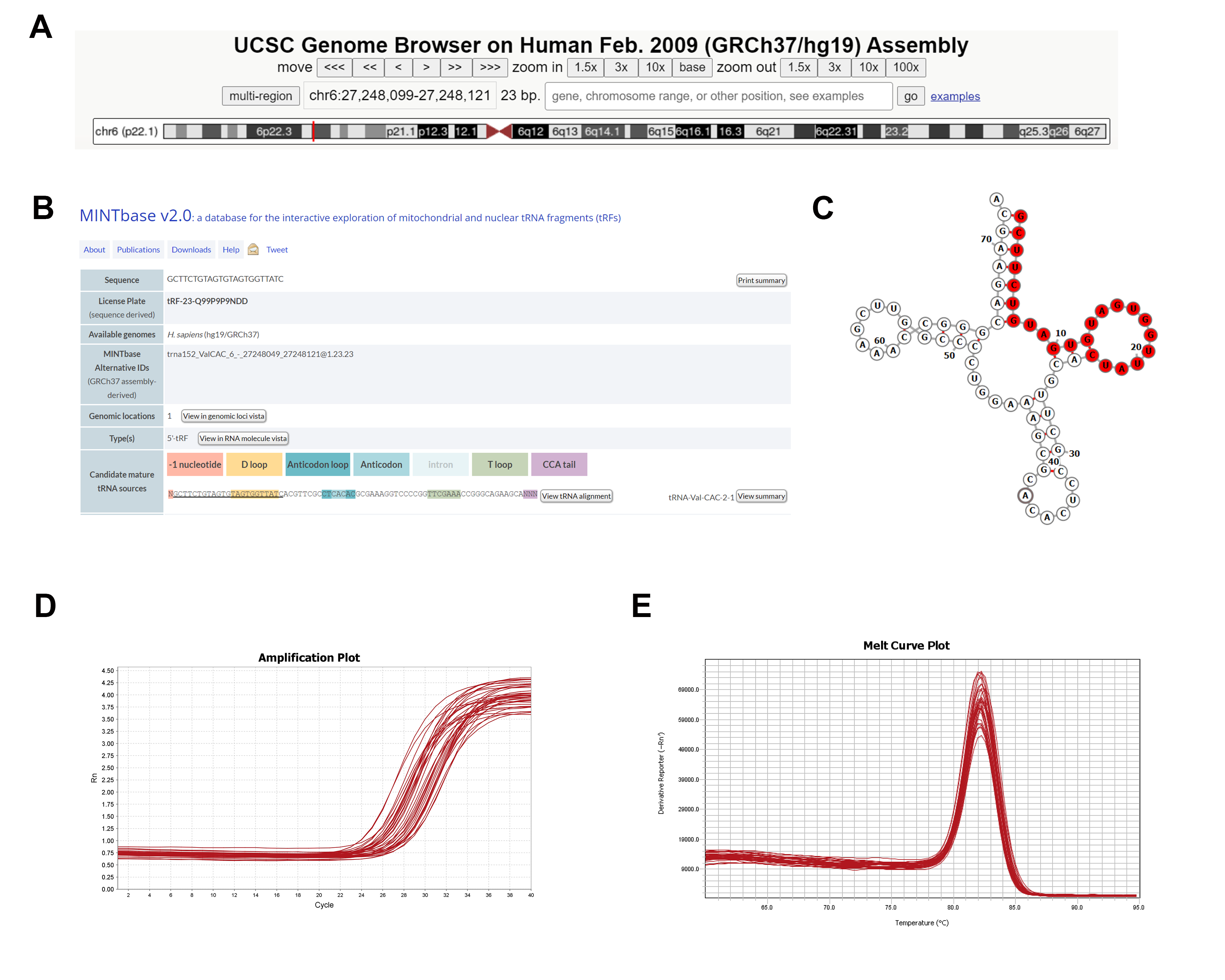

Supplement: Supplementary file 1 — Additional file 1: Fig. S1. tRF-23-Q99P9P9NDD is a sortof tRFs. A UCSC Genome Browser database showed that tRF-23-Q99P9P9NDDwas located on chromosome 6p22.1 with coordinates 27,248,099–27,248,121. B InMINTbase v2.0, tRF-23-Q99P9P9NDD is a 5′tRF (GCTTCTGTAGTGTAGTGGTTATC) with alength of 23nt. C OncotRF database showed that the cleavage site waslocated on the D-loop. D Amplification curves of tRF-23-Q99P9P9NDD. EMelting curves of tRF-23-Q99P9P9NDD. [file 10020_2022_491_MOESM1_ESM.tif]

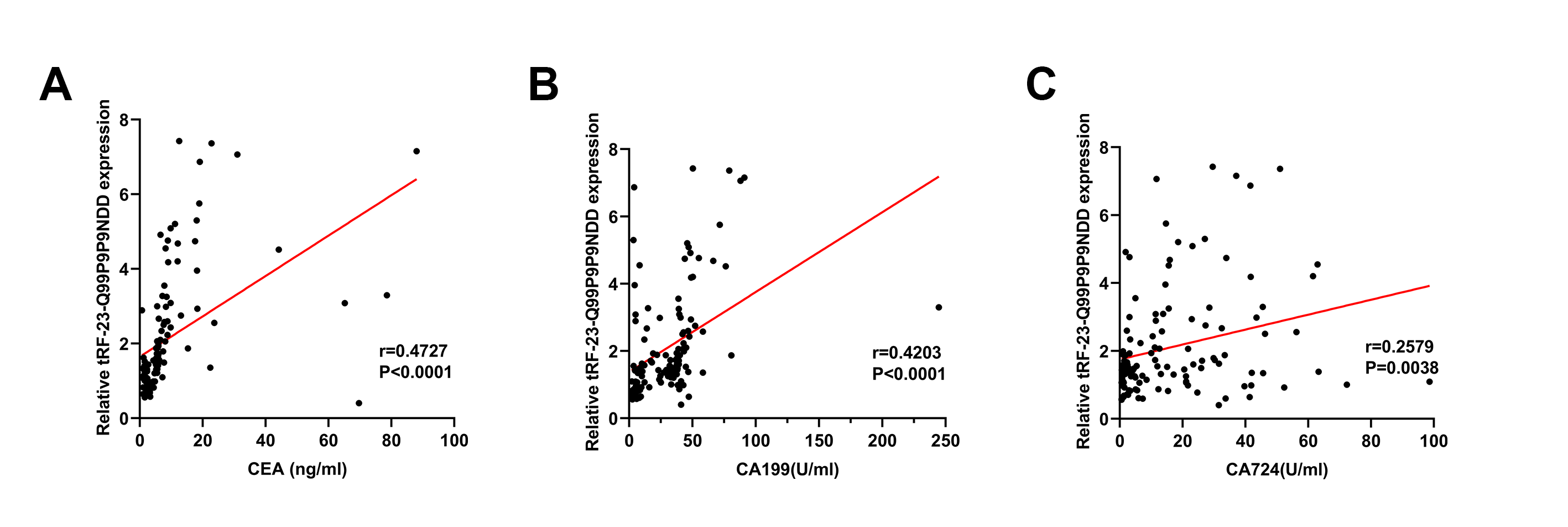

Supplement: Supplementary file 2 — Additional file 2: Fig. S2. The correlation analysis between the expression levels of serumtRF-23-Q99P9P9NDD and conventional biomarkers of GC. A-C Pearsoncorrelation analysis was performed on the expression level of tRF-23-Q99P9P9NDDand the levels of CEA, CA199, and CA724 in GC serum. [file 10020_2022_491_MOESM2_ESM.tif]
