# Supplementary material for: Evaluation of serum tRF-23-Q99P9P9NDD as a potential biomarker for the clinical diagnosis of gastric cancer
Source: Mol Med. 2022 Jun 11;28:63. doi: 10.1186/s10020-022-00491-8 (PMC9188071; doi:10.1186/s10020-022-00491-8)
Supplement: Supplementary file 3 — Additional file 3: Table S1. ROC analysis of allbiomarkers in distinguishing GC patients from healthy donors. [file 10020_2022_491_MOESM3_ESM.docx]

**Table S1** **ROC analysis of all biomarkers in distinguishing GC patients from healthy donors**

|  | AUC | P-value | 95% confidence interval (CI) |  |
| --- | --- | --- | --- | --- |
|  |  |  |  |  |
| tRF-23-Q99P9P9NDD | 0.783 | <0.0001 | 0.724-0.842 |  |
| CEA | 0.715 | <0.0001 | 0.649-0.779 |  |
| CA199 | 0.614 | 0.002 | 0.543-0.685 |  |
| CA724 | 0.751 | <0.0001 | 0.690-0.812 |  |
| tRF-23-Q99P9P9NDD+CEA | 0.789 | <0.0001 | 0.731-0.847 |  |
| tRF-23-Q99P9P9NDD+CA199 | 0.797 | <0.0001 | 0.739-0.854 |  |
| tRF-23-Q99P9P9NDD+CA724 | 0.845 | <0.0001 | 0.794-0.895 |  |
| tRF-23-Q99P9P9NDD+CEA+CA199 | 0.812 | <0.0001 | 0.756-0.866 |  |
| tRF-23-Q99P9P9NDD+CEA+CA724 | 0.846 | <0.0001 | 0.795-0.896 |  |
| tRF-23-Q99P9P9NDD+CA199+CA724 | 0.853 | <0.0001 | 0.803-0.902 |  |
| tRF-23-Q99P9P9NDD+CEA+CA199+CA724 | 0.862 | <0.0001 | 0.815-0.908 |  |
