# Supplementary material for: Evaluation of serum tRF-23-Q99P9P9NDD as a potential biomarker for the clinical diagnosis of gastric cancer
Source: Mol Med. 2022 Jun 11;28:63. doi: 10.1186/s10020-022-00491-8 (PMC9188071; doi:10.1186/s10020-022-00491-8)
Supplement: Supplementary file 4 — Additional file 4: Table S2. ROC analysis of allbiomarkers in distinguishing early GC patients from healthy donors. [file 10020_2022_491_MOESM4_ESM.docx]

**Table S2 ROC analysis of all biomarkers in distinguishing early GC patients from healthy donors**

|  | AUC | P-value | 95% confidence interval (CI) |  |
| --- | --- | --- | --- | --- |
|  |  |  |  |  |
| tRF-23-Q99P9P9NDD | 0.724 | <0.0001 | 0.648-0.800 |  |
| CEA | 0.664 | <0.0001 | 0.584-0.743 |  |
| CA199 | 0.590 | 0.031 | 0.506-0.673 |  |
| CA724 | 0.711 | <0.0001 | 0.636-0.787 |  |
| tRF-23-Q99P9P9NDD+CEA | 0.732 | <0.0001 | 0.657-0.807 |  |
| tRF-23-Q99P9P9NDD+CA199 | 0.741 | <0.0001 | 0.666-0.816 |  |
| tRF-23-Q99P9P9NDD+CA724 | 0.798 | <0.0001 | 0.731-0.864 |  |
| tRF-23-Q99P9P9NDD+CEA+CA199 | 0.760 | <0.0001 | 0.689-0.831 |  |
| tRF-23-Q99P9P9NDD+CEA+CA724 | 0.802 | <0.0001 | 0.736-0.869 |  |
| tRF-23-Q99P9P9NDD+CA199+CA724 | 0.805 | <0.0001 | 0.739-0.871 |  |
| tRF-23-Q99P9P9NDD+CEA+CA199+CA724 | 0.819 | <0.0001 | 0.756-0.881 |  |
