# Supplementary material for: Evaluation of serum tRF-23-Q99P9P9NDD as a potential biomarker for the clinical diagnosis of gastric cancer
Source: Mol Med. 2022 Jun 11;28:63. doi: 10.1186/s10020-022-00491-8 (PMC9188071; doi:10.1186/s10020-022-00491-8)
Supplement: Supplementary file 5 — Additional file 5: Table S3. ROC analysis of all biomarkers indistinguishing GC patients from gastritis patients. [file 10020_2022_491_MOESM5_ESM.docx]

**Table S3 ROC analysis of all biomarkers in distinguishing GC patients from gastritis patients**

|  | AUC | P-value | 95% confidence interval (CI) |  |
| --- | --- | --- | --- | --- |
|  |  |  |  |  |
| tRF-23-Q99P9P9NDD | 0.685 | <0.0001 | 0.605-0.766 |  |
| CEA | 0.678 | <0.0001 | 0.594-0.761 |  |
| CA199 | 0.605 | 0.031 | 0.512-0.697 |  |
| CA724 | 0.676 | <0.0001 | 0.594-0.757 |  |
| tRF-23-Q99P9P9NDD+CEA | 0.696 | <0.0001 | 0.616-0.777 |  |
| tRF-23-Q99P9P9NDD+CA199 | 0.705 | <0.0001 | 0.626-0.785 |  |
| tRF-23-Q99P9P9NDD+CA724 | 0.745 | <0.0001 | 0.670-0.820 |  |
| tRF-23-Q99P9P9NDD+CEA+CA199 | 0.716 | <0.0001 | 0.637-0.795 |  |
| tRF-23-Q99P9P9NDD+CEA+CA724 | 0.747 | <0.0001 | 0.672-0.822 |  |
| tRF-23-Q99P9P9NDD+CA199+CA724 | 0.758 | <0.0001 | 0.685-0.832 |  |
| tRF-23-Q99P9P9NDD+CEA+CA199+CA724 | 0.764 | <0.0001 | 0.690-0.837 |  |
